# Deep Learning-Based Prediction Model of Surgical Indication of Nasal Bone Fracture Using Waters’ View

**DOI:** 10.3390/diagnostics15182386

**Published:** 2025-09-19

**Authors:** Dong Yun Lee, Soo A Lim, Su Rak Eo

**Affiliations:** Department of Plastic and Reconstructive Surgery, Dongguk University Ilsan Hospital, Dongguk University College of Medicine, Seoul 10326, Republic of Korea; psdylee@gmail.com (D.Y.L.);

**Keywords:** deep learning, nasal bone fracture, Waters’ view, surgical indication, artificial intelligence

## Abstract

**Background/Objectives:** The nasal bone is critical to both the functional integrity and esthetic contour of the facial skeleton. Nasal bone fractures constitute the most prevalent facial fracture presentation in emergency departments. The identification of these fractures and the determination of immediate intervention requirements pose significant challenges for inexperienced residents, potentially leading to oversight. **Methods:** A retrospective analysis was conducted on facial trauma patients undergoing cranial radiography (Waters’ view) during initial emergency department assessment between March 2008 and July 2022. This study incorporated 2099 radiographic images. Surgical indications comprised the displacement angle, interosseous gap size, soft tissue swelling thickness, and subcutaneous emphysema. A deep learning-based artificial intelligence (AI) algorithm was designed, trained, and validated for fracture detection on radiographic images. Model performance was quantified through accuracy, precision, recall, and F1 score. Hyperparameters included the batch size (20), epochs (70), 50-layer network architecture, Adam optimizer, and initial learning rate (0.001). **Results:** The deep learning AI model employing segmentation labeling demonstrated 97.68% accuracy, 82.2% precision, 88.9% recall, and an 85.4% F1 score in nasal bone fracture identification. These outcomes informed the development of a predictive algorithm for guiding conservative versus surgical management decisions. **Conclusions:** The proposed AI-driven algorithm and criteria exhibit high diagnostic accuracy and operational efficiency in both detecting nasal bone fractures and predicting surgical indications, establishing its utility as a clinical decision-support tool in emergency settings.

## 1. Introduction

### 1.1. Research Objective

The nose is a paramount esthetic feature of the human face, with nasal bone fractures ranking as the most prevalent bone injury in adults and the third most common fracture across the entire body [1]. Approximately 40% of facial trauma incidents involve fractures of the nasal bones, accounting for approximately 50,000 cases annually in the United States alone [1,2]. Despite the perception that nasal bone fractures are minor injuries, prompt and precise diagnosis is crucial [3]. Delayed treatment may result in functional and cosmetic complications, exacerbating traumatic edema, preexisting nasal deformities, and occult septal injuries [4,5]. Consequently, promptly and swiftly achieving an accurate diagnosis determines the need for surgical intervention, which plays a pivotal role in ensuring favorable patient outcomes [3,4].

Our study introduces a predictive model based on deep learning techniques, aiming to provide healthcare practitioners with a diagnostic tool that enhances the efficiency and effectiveness of managing patients with nasal trauma visiting the emergency department. Moreover, our model sought to expedite the determination of surgical indications and streamline the decision-making process within a compressed timeframe.

### 1.2. Research Scope and Methods

We performed a retrospective review of all patients with facial trauma who underwent occipitomental radiography (Waters’ view) at their initial visit to the emergency room between March 2008 and July 2022.

### 1.3. Research Scope

**Figure 1:**
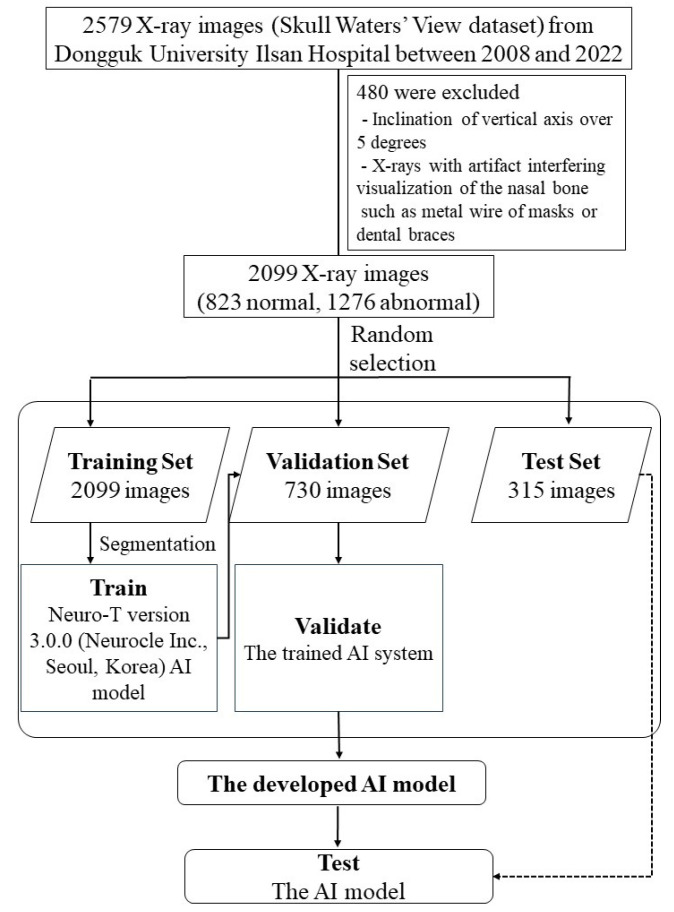
A flowchart of image selection process.

A dataset consisting of 2579 skull Waters’ view radiographs was collected with the approval of the individual institutional review board (IRB no. 2023-05-017). A total of 2099 radiographs were used in this study. Patients treated surgically for nasal bone fractures at the Department of Plastic and Reconstructive Surgery of Dongguk University Ilsan Hospital were included. We excluded 480 radiographs due to issues including an inclination of the vertical axis over 5° and obvious artifacts interfering with the visualization of the nasal bone, such as metal wires of masks or dental braces, were excluded. The total number of images comprised 823 normal and 1276 fracture radiographs. The training, validation, and test sets were randomly selected from the finalized cohort of 2099 samples, resulting in training, validation, and test cohorts consisting of 2099, 730, and 315 patients, respectively (Figure 1).

### 1.4. Patients and Methods

We identified the surgical indication factors, including displacement angle, size of the bony gap, thickness of soft tissue swelling, and existence of subcutaneous emphysema. Two experienced clinicians determined the presence or absence of nasal bone fractures. The patients who underwent closed reduction were included in the fracture group. An AI algorithm based on deep learning was engineered, trained, and validated to detect fractures in radiographs.

### 1.5. Automated Deep Learning Tool for Model Establishment

A no-code deep learning tool was used in this study. Neuro-T version 3.0.0 (Neurocle Inc., Seoul, Republic of Korea) automatically builds effective deep convolutional neural networks (DCNN) for image recognition and classification using a software algorithm that analyzes the features of the dataset and self-discovers optimal hyperparameters, thus making it easy for non-experts to build the best models.

The hardware system for establishing the Neuro-T-based model included four GeForce RTX 2080 Ti GPUs, dual Intel^®^ Xeon CPUs, and 256 GB RAM.

**Figure 2:**
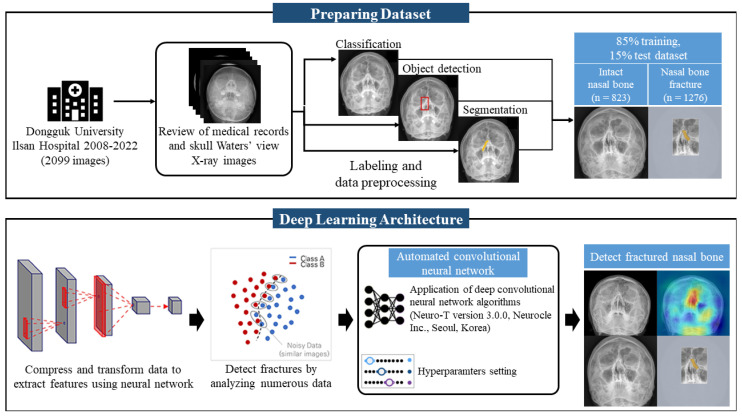
Schematic Illustration of Convolutional Neural Network (CNN) Applications. The training process centered around a single CNN architecture for the purpose of comparing accuracy performance in the detection of nasal bone fractures, according to the labeling methods applied to the dataset. In the ‘Preparing Dataset’ section of the figure, the fracture site indicated by clinicians is marked with a red box for object detection labeling. In the ‘Deep Learning Architecture’ section, the red box illustrates the process of data compression and transformation for feature extraction using a neural network.

Figure 2 illustrates a schematic representation of convolutional neural network (CNN) applications. The dataset was curated from anonymized raw skull Waters’ view radiographic images, which were meticulously cropped and labeled for the study. The training process centered around a single CNN architecture for the purpose of comparing accuracy performance in the detection of nasal bone fractures, according to the labeling methods applied to the dataset.

### 1.6. Hyperparameter

In the context of the hyperparameter settings for our experiment, a batch size of 20 and 70 epochs, a network architecture consisting of 50 layers, utilization of the Adam optimizer, and an initial learning rate of 0.001 were employed.

## 2. Theoretical Background

### 2.1. Diagnosis of Nasal Bone Fracture

Conventional diagnostic approaches for nasal bone fractures typically rely on physical examinations and the clinical expertise of healthcare professionals [3]. Although clinical symptoms may raise suspicion of nasal fractures, they often require confirmation through facial bone computed tomography (CT) to ascertain both the accurate diagnosis and extent of nasal deformity [3,4,5]. Nevertheless, these conventional methods suffer from potential drawbacks such as time-intensive procedures, subjectivity, and susceptibility to inter-observer variability [3]. These limitations can lead to delayed diagnosis and suboptimal treatment outcomes. In particular, in Korea, emergency medicine practitioners, who are responsible for initiating primary radiologic diagnostic assessments for patients with facial trauma, serve a diverse patient population, thus encountering challenges in diagnosing nasal bone fractures through radiography or selecting the appropriate facial bone CT in a timely manner [1,2,3].

### 2.2. Artificial Intelligence (AI) in Fracture Diagnosis

Recent advances in artificial intelligence (AI) have yielded promising results in the analysis and prognostication of medical imaging data [6,7,8,9]. In this study, we presented an innovative AI-based predictive algorithm tailored to assess the surgical necessity for nasal bone fractures by leveraging Waters’ view radiographs. The Waters’ view constitutes a standard radiographic projection meticulously designed to visualize the nasal bones and contiguous anatomical structures [2,3]. Existing research has primarily focused on lateral-view radiographs and deep learning, or convolutional neural network (CNN) methodologies applied to three-dimensional reconstructed CT images [10,11,12,13]. While lateral view radiographs are notably beneficial for the diagnosis and pre- and postoperative assessment of nasal tip fractures, their efficacy in diagnosing unilateral nasal bone fractures, fractures involving deviations, and similar cases is notably limited when relying solely on radiographs [12].

## 3. Results

### 3.1. Patients

Baseline characteristics of the study cohort are summarized in Table 1. The training, validation, and test cohorts consisted of 2099, 730, and 315 patients, respectively (mean age, 38–41 years; male, 55.9–63.3%; fractures, 54.9–60.8%). There were significantly more males in the training and validation cohorts (62.7–63.3%) than in the test sets (55.9%) (*p*  <  0.001). There were no significant differences among the cohorts in mean age (*p*  =  0.112) or proportion of fractures (*p*  =  0.081).

### 3.2. Diagnostic Performance of Deep Learning Model

**Figure 3:**
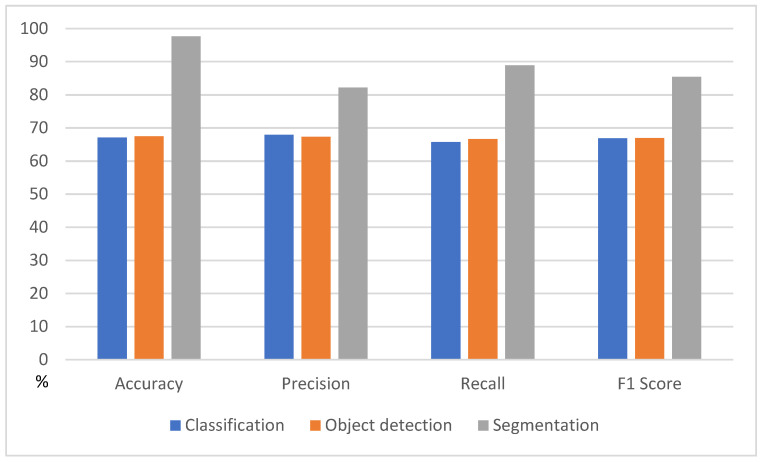
Graph comparing the fracture detection performance of AI models by labeling method.

The diagnostic performance measures of the model with the classification method showed 67.09% accuracy, 67.90% precision, 65.80% recall, and an F1 score of 66.80%. The diagnostic performance measures of the model with the object-detection method showed 67.41% accuracy, 67.30% precision, 66.7% recall, and an F1 score of 67.00%. The deep learning model with segmentation labeling demonstrated excellent diagnostic performance, with 97.68% accuracy, 82.2% precision, 88.9% sensitivity, and an 85.4% F1 score (Table 2). Figure 3 visualizes the significantly superior performance of the segmentation labeling method compared to classification and object detection. Furthermore, the diagnostic performance measures of the deep learning model with segmentation labeling and quick learning showed 97.34% accuracy, 79.8% precision, 89.4% recall, and 84.3% F1 score. The dataset with the region of interest (ROI) application for the nasal bone region and segmentation of the fractured area amplified the accuracy of the fracture diagnosis. The result was significantly improved and more accurate than that of the classification or object detection labeling method.

**Figure 4:**
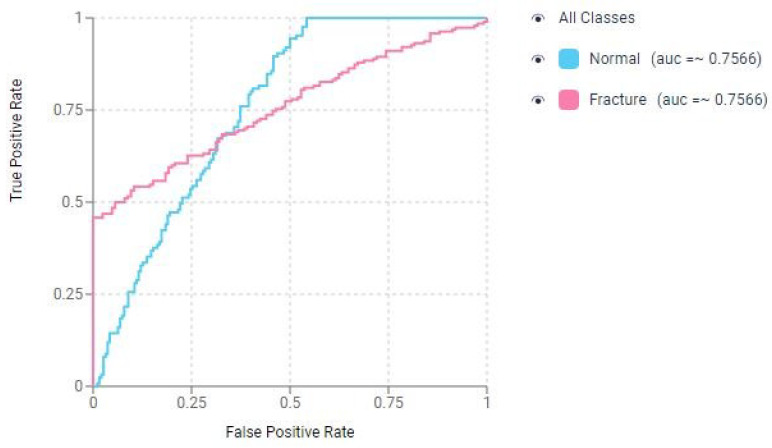
Receiver operating characteristic (ROC) curve of the convolutional neural network (CNN) for detecting nasal bone fractures in the prediction model with classification.

Figure 4 displays the Receiver Operating Characteristic (ROC) curve for the CNN in the classification-based prediction model for nasal bone fracture detection.

**Figure 5:**
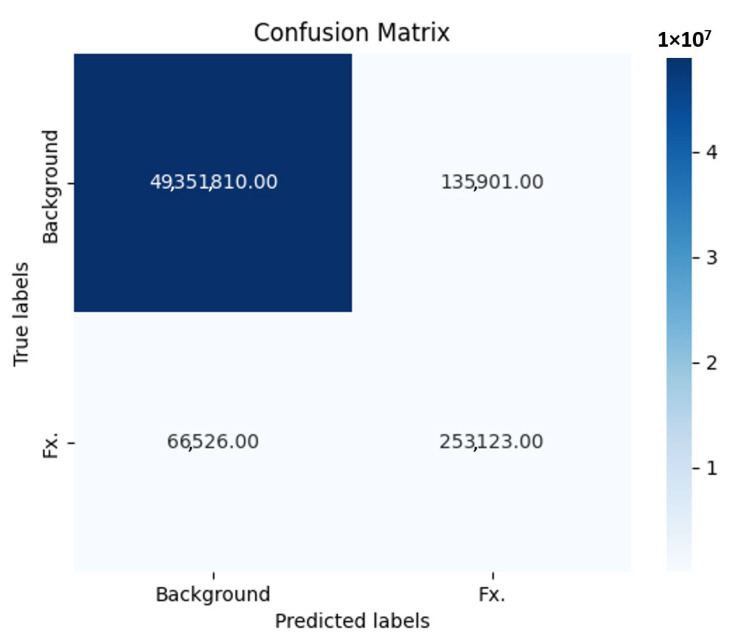
The key performance metrics of our fracture detection model with segmentation labeling are presented in the form of a confusion matrix. These metrics were calculated based on the following values: 253,123 true positive cases, 66,526 false negative cases, 49,351,810 true negative cases, and 135,901 false positive cases. Our model exhibited a notable level of sensitivity, approximately 88.9%. The specificity was calculated using true negatives and false positives with a value of 99.73%.

The key performance metrics of our fracture detection model with segmentation labeling are presented in the form of a confusion matrix (Figure 5). These metrics were calculated based on the following values: 253,123 true positive cases, 66,526 false negative cases, 49,351,810 true negative cases, and 135,901 false positive cases. Our model exhibited a notable level of sensitivity, approximately 0.889, signifying its ability to accurately identify positive cases, or fractures, among the total instances. Additionally, the model demonstrated a high degree of specificity, approximately 0.9973, indicating its proficiency in correctly classifying negative cases, or non-fractures. The Negative Predictive Value (NPV) achieved a rate of 99.87%.

**Figure 6:**
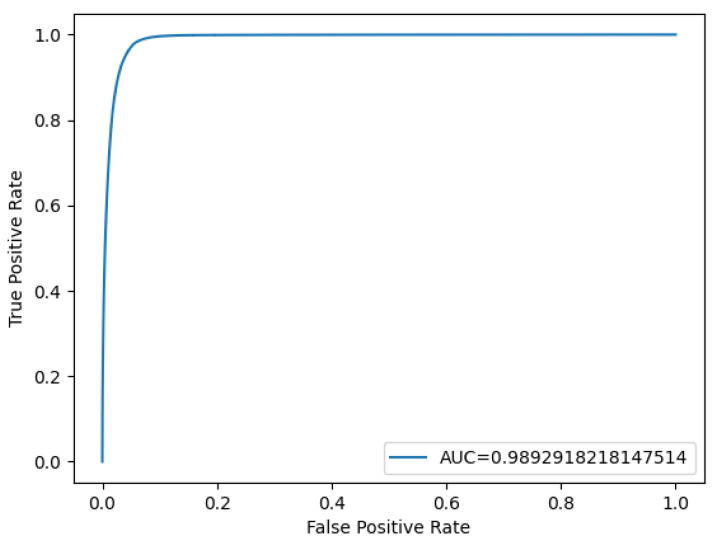
Receiver operating characteristic (ROC) curve of the convolutional neural network (CNN) for detecting nasal bone fractures in the prediction model with segmentation labeling.

Based on a sensitivity of 88.9% and a specificity of 99.73%, a ROC curve and an Area Under the Curve (AUC) score were generated using the probability values from both the ground truth images and the model’s results (Figure 6). The AUC value was calculated as 0.99 (rounded to two decimal places). These results emphasize the model’s robustness and reliability in accurately detecting fractures while minimizing false positives, making it a promising tool for medical applications.

### 3.3. True Positive and False Positive of Deep Learning Models

**Figure 7:**
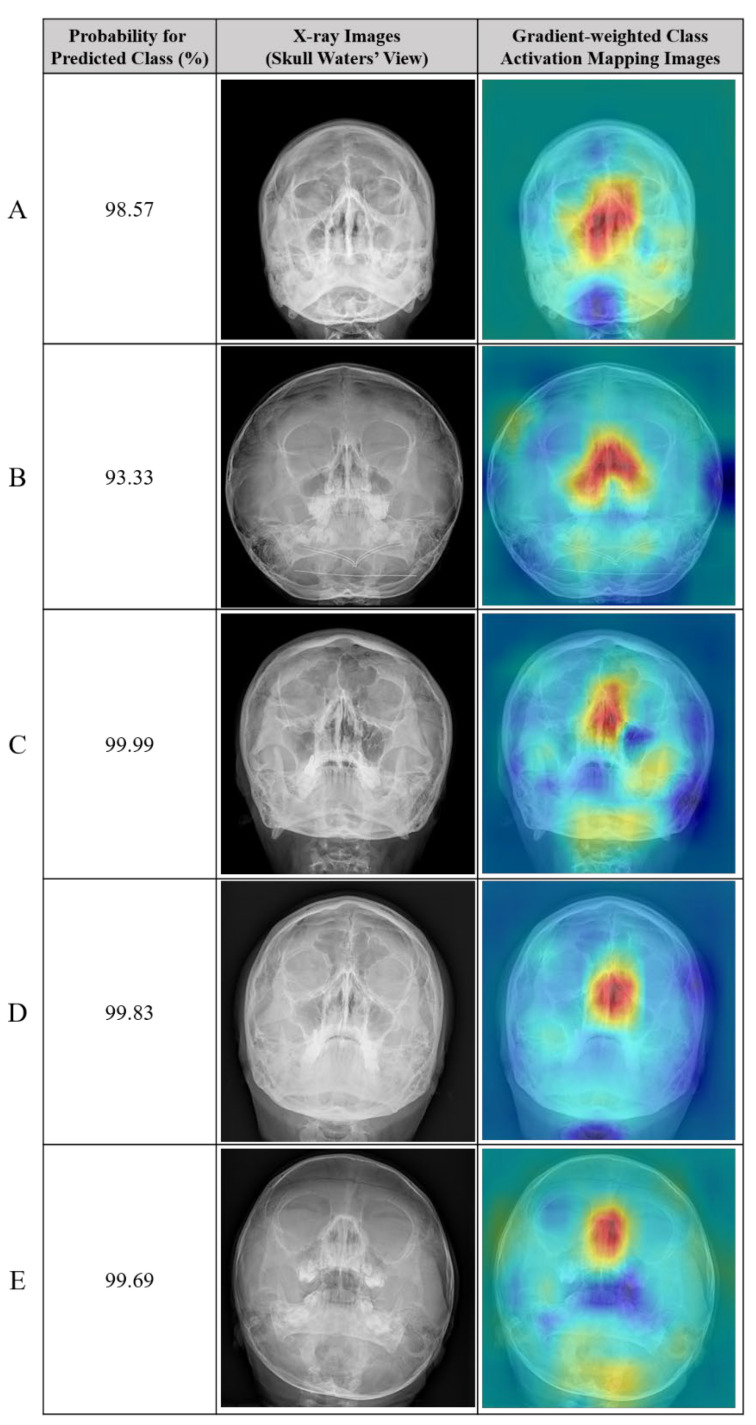
Representative cases of correctly predicted classes (true positive) in the test dataset using the no-code platform with a classification labeling method. (**Left**) Radiographic images of the Skull Waters’ view with the removal of right/left markings from the corners of the X-ray. (**Right**) Gradient-weighted class activation mapping (CAM) images. The probability for the predicted class (%) is also provided for each sample. (**A**–**D**) fractures of the unilateral nasal side wall; (**E**) fracture of the bilateral nasal side walls. In the gradient-weighted class activation maps (CAM) image for case (**E**), the background appears lighter because the neural network assigns lower activation to these regions, indicating reduced importance for the classification decision.

**Figure 8:**
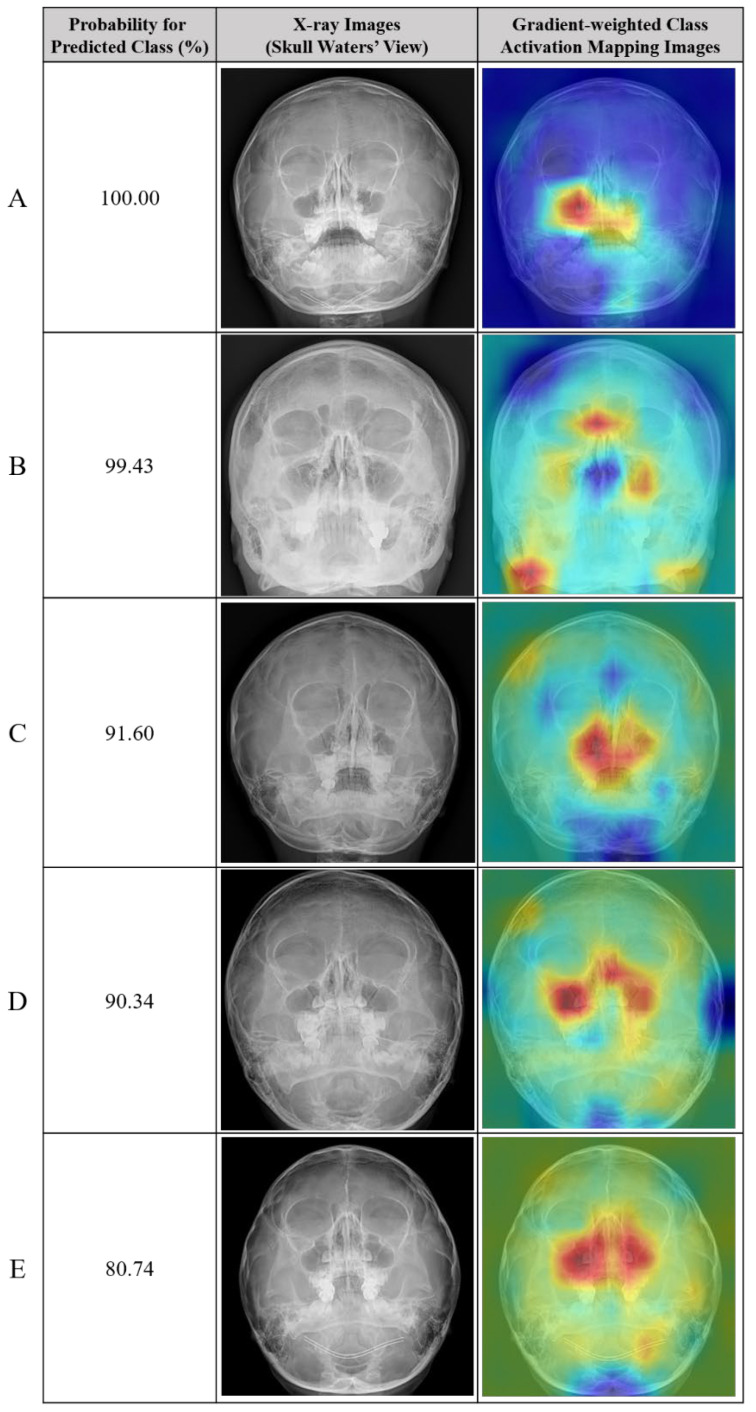
Representative cases of incorrectly predicted classes (false positive) in the test dataset using the no-code platform with a classification labeling method. (**Left**) Radiographic images of the Skull Waters’ view with the removal of right/left markings from the corners of the X-ray. (**Right**) Gradient-weighted class activation mapping (CAM) images. The probability for the predicted class (%) is also provided for each sample. (**A**–**E**) normal nasal radiographs misclassified as fractures.

The accuracy and F1 score of the deep learning model with segmentation labeling were significantly higher than those of the classification model (85.4% and 66.8%, respectively). Example radiographs of nasal bone fractures correctly diagnosed using gradient-weighted class activation maps (CAM) in the classification model are shown in Figure 7. The nasal bone area is highlighted in the heat map of the X-rays with correct detection. In addition, examples of normal nasal radiographs incorrectly diagnosed as fractures using the deep learning model with classification are shown in Figure 8. The probability for the predicted class (%) is also provided for each sample.

**Figure 9:**
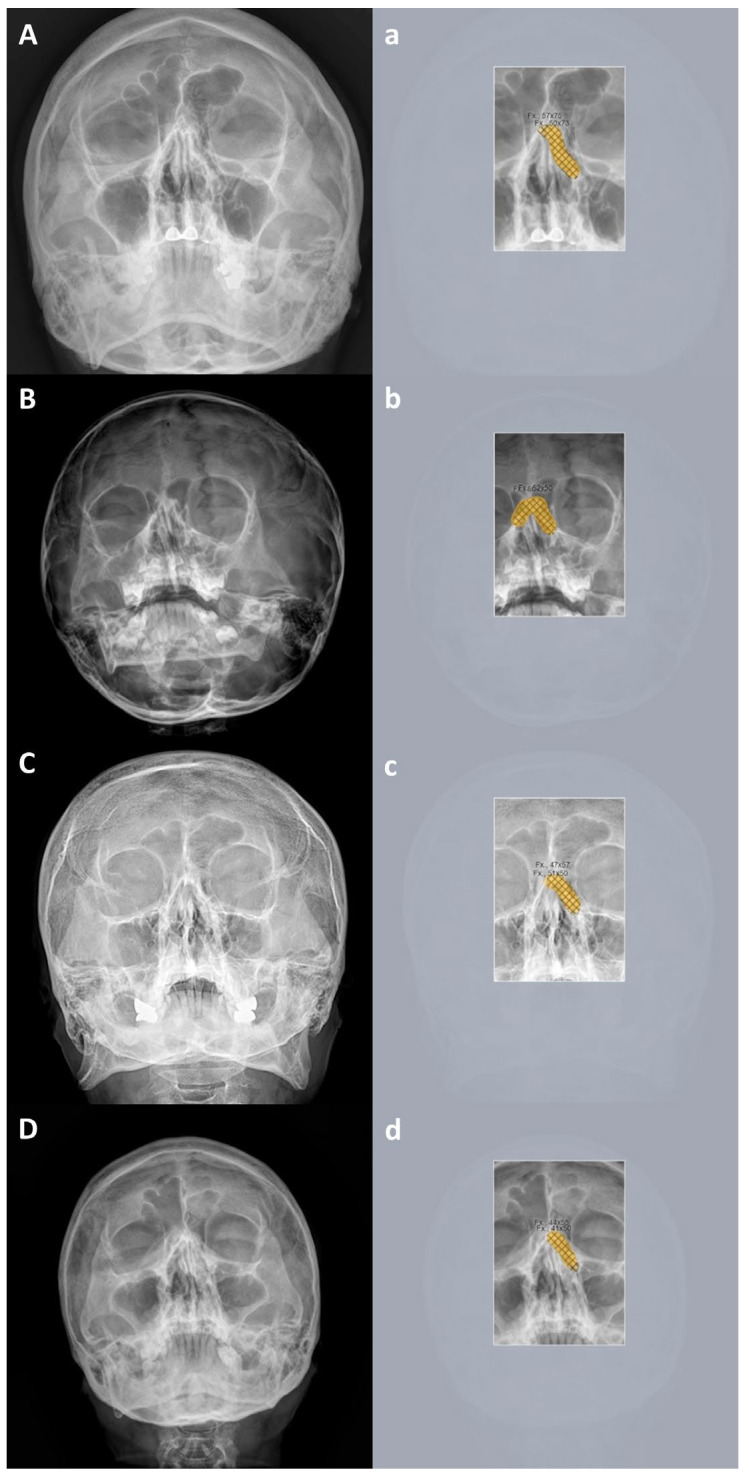
Example images of skull waters’ view radiographs overlaid with segmentation labeling with correct detection. The deep-learning model correctly identified the nasal bone fractures. (**Left**) Radiographic images of the Skull Waters’ view with the removal of right/left markings from the corners of the X-ray. (**Right**) The orange color represents the area labeled by the authors using a segmentation method, while the hatched part corresponds to the pixel area predicted as fractures by the deep-learning model. The images (**A**,**a**), (**B**,**b**) and (**C**,**c**) show typical unilateral nasal bone fractures with fracture lines; images (**D**,**d**) show comminuted fractures with soft tissue swelling.

**Figure 10:**
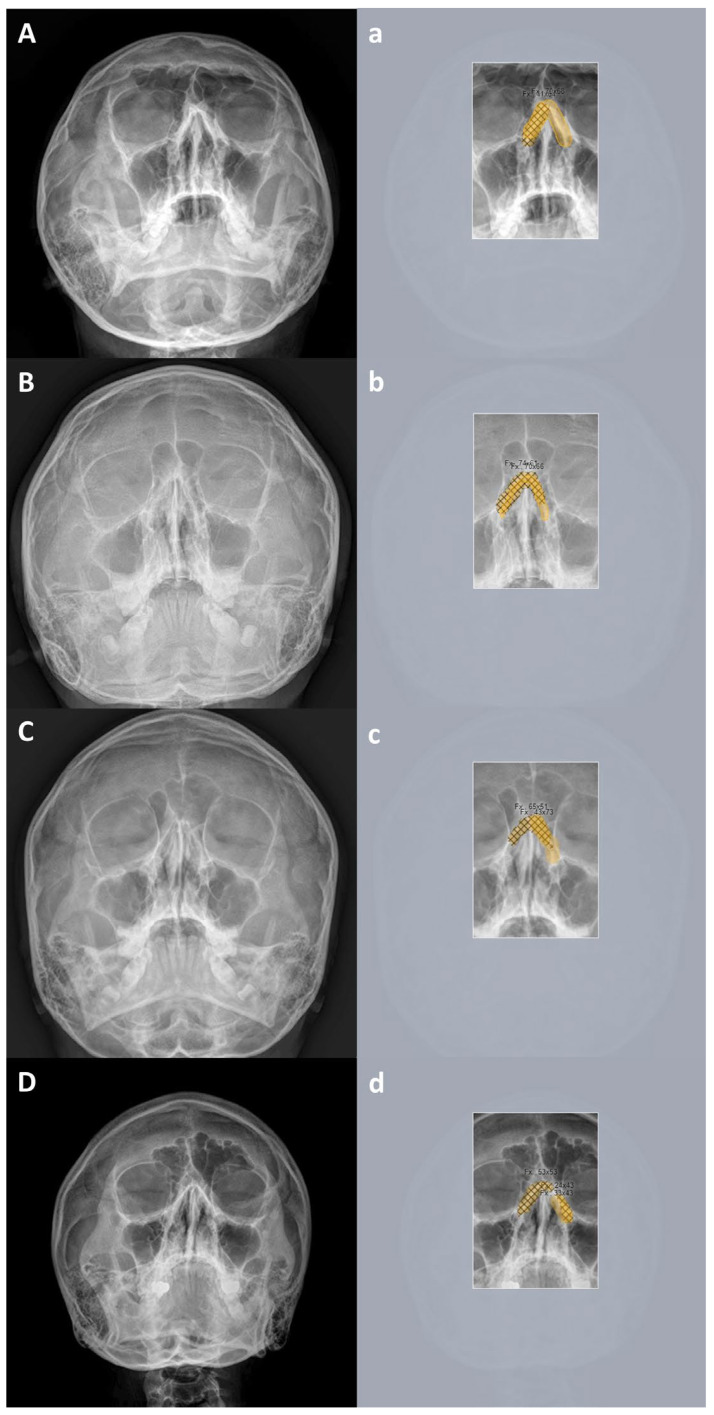
Representative cases of incorrectly predicted classes in the test dataset using the no-code platform with a segmentation labeling method. (**Left**) Radiographic images of the Skull Waters’ view with the removal of right/left markings from the corners of the X-ray. (**Right**) The orange color represents the area labeled by the authors using a segmentation method, while the hatched part corresponds to the pixel area predicted as fractures by the deep-learning model. The images (**A**,**a**), (**B**,**b**), (**C**,**c**) and (**D**,**d**) show normal nasal radiographs misclassified as fractures.

Example images of skull waters’ view radiographs overlaid with the segmentation labeling with correct detection are exhibited in (Figure 9). The deep-learning model correctly identified the nasal bone fractures. Figure 10 displays representative cases of incorrectly predicted classes in the test dataset using the no-code platform with the segmentation labeling method. The orange color represents the area labeled by the authors using a segmentation method, while the hatched part corresponds to the pixel area predicted as fractures by the deep-learning model. Based on these results, we proposed a prediction algorithm to determine whether conservative treatment or surgical intervention should be performed.

## 4. Discussion

The nose is the foremost esthetic focal point of the human face, and nasal bone fractures constitute the most prevalent bone injuries in adults, ranking as the third most common fracture across the entire body [1,2]. Facial trauma incidents involving nasal bone fractures are estimated to comprise approximately 40% of all cases, translating into approximately 50,000 occurrences annually in the United States alone [1]. Although nasal bone fractures are often regarded as minor injuries, expeditious and accurate diagnosis of these fractures is of paramount significance [3,4,5]. This is primarily because delayed treatment has been linked to functional and cosmetic complications, the exacerbation of traumatic edema, preexisting nasal deformities, and occult septal injuries [4]. Consequently, promptly and swiftly achieving an accurate diagnosis determines the need for surgical intervention, which plays a pivotal role in ensuring favorable patient outcomes [5].

Traditional diagnostic approaches for nasal bone fractures typically rely on physical examinations and the clinical expertise of healthcare professionals [2]. Although clinical symptoms may raise suspicion of nasal fractures, they often require confirmation through facial bone computed tomography (CT) to ascertain both the accurate diagnosis and extent of nasal deformity [3]. Nevertheless, these conventional methods suffer from potential drawbacks such as time-intensive procedures, subjectivity, and susceptibility to inter-observer variability [3,4,5]. These limitations can lead to delayed diagnosis and suboptimal treatment outcomes. In particular, in Korea, emergency medicine practitioners, who are responsible for initiating primary radiologic diagnostic assessments for patients with facial trauma, serve a diverse patient population, thus encountering challenges in diagnosing nasal bone fractures through radiography or selecting the appropriate facial bone CT in a timely manner.

Recent advances in the field of AI have yielded promising results in the analysis and prognostication of medical imaging data [14,15,16,17,18,19,20,21]. In this study, we present an innovative AI-based predictive algorithm tailored to assess the surgical necessity for nasal bone fractures by leveraging Waters’ view radiographs. The Waters’ view constitutes a standard radiographic projection meticulously designed to visualize the nasal bones and contiguous anatomical structures [2,3,22]. To date, existing research has primarily focused on lateral-view radiographs, and deep learning or CNN methodologies applied to three-dimensional reconstructed CT images [10,11,12,13]. While lateral view radiographs are notably beneficial for the diagnosis and pre- and postoperative assessment of nasal tip fractures, their efficacy in diagnosing unilateral nasal bone fractures, fractures involving deviations, and similar cases is notably limited when relying solely on radiographs [2]. Our study introduces a predictive model based on deep learning techniques, aiming to provide healthcare practitioners with a diagnostic tool that enhances the efficiency and effectiveness of managing patients with nasal trauma visiting the emergency department. Moreover, our model sought to expedite the determination of surgical indications and streamline the decision-making process within a compressed timeframe.

With advancements in artificial intelligence systems, active research has been conducted in the craniomaxillofacial area, but not specifically on solitary nasal bone fractures using Waters’ view [23,24,25,26,27,28,29]. Recent studies have demonstrated significant progress in deep learning for orthopedic imaging, while its application to facial fractures remains limited, with 2025 reports highlighting ongoing work on the diagnosis and surgical indications of orbital fractures [30,31]. A limited number of publications have also addressed orthognathic surgery [23], cosmetic instrumentation using 3-D facial images [6,7,8], and dental image analysis, including implant dentistry [24]. AI research on facial bone fractures has been largely based on 3-D reconstructed CT) images [10,11,12,13]. Even when radiography is used, only a few studies have used the nasal lateral view for nasal bone fractures [12], and there have been more studies on the zygoma complex [6].

In this study, we proposed a novel AI-based prediction algorithm for the surgical indication of nasal bone fractures using Waters’ view radiographs. Our algorithm uses a convolutional neural network (CNN) to analyze radiographs and predict whether surgical intervention is necessary in patients with nasal bone fractures. We trained and validated our algorithm using a dataset of 2099 patients with nasal bone fractures, who underwent surgical or conservative treatment. The results showed that our AI algorithm achieved high accuracy and reliability, with a sensitivity of 88.9%, a specificity of 99.73%, and an overall accuracy of 97.68%. These results are comparable to those of studies on the diagnosis of nasal bone fractures using machine learning techniques with other radiological modalities [10,11,12].

Moreover, by utilizing Waters’ view and AI to identify nasal bone fractures, we gained several additional advantages beyond the previously mentioned time efficiency. This mitigates unnecessary labor costs and benefits national finances in terms of health insurance. In South Korea, where the health insurance system continues to face deficit problems, a 3-D facial bone CT scan costs approximately $335.5, but the national insurance covers $239.6; therefore, the more unnecessary the CT scan, the more it costs the country. However, Waters’ view radiographs cost approximately $13.2, and the government covers $9.4, which is significantly less than the cost of a CT scan, thereby substantially reducing the national financial burden per person [1,2,32].

Furthermore, there are evident health benefits for patients. A typical skull radiograph imparts an effective radiation dose of 0.1 mSv, while a CT scan imparts an effective radiation dose of 10 mSv, signifying a considerable disparity. Radiographs dramatically diminish radiation exposure in patients [32].

Although our study demonstrated the potential of AI in the diagnosis and prediction of nasal bone fractures, some limitations need to be addressed. First, our dataset was relatively small and was collected from a single institution, which may have limited the generalizability of our findings. The lack of external validation was the most significant limitation. It cannot be excluded that the diagnostic accuracy would be low if data from other radiograph machines at other institutions were used to confirm the diagnosis. Further studies, using larger datasets from multiple institutions, are required to verify our results. Second, our algorithm was trained and validated using only one type of radiograph from the Waters’ view. Additional studies are required to determine the generalizability of our algorithm to other types of radiographs. Third, the study samples were biased toward simple nasal bone fractures, which may have led to potential overestimation of model performance. The abnormal group included only isolated nasal bone fractures, while complex cases such as those involving the orbital rim/wall or zygomaticomaxillary complex were excluded as they could interfere with deep learning training. However, isolated nasal bone fractures are considerably more common than complex facial fractures in emergency settings [1,2], where complex cases typically undergo CT scans due to severity and surgical requirements. Finally, although bilateral nasal bones can be identified, fractures of the nasal tip are difficult to detect. To overcome this limitation, further multicenter studies, secure external validation, and engineering techniques should be developed to filter and distinguish imaging noise more accurately.

## 5. Conclusions

In conclusion, our study provides a new basis for the efficient diagnosis of nasal bone fractures by interpreting Waters’ view images using AI. This diagnostic prediction model speeds up diagnoses in the emergency department and helps less-experienced doctors order, read, and diagnose imaging tests. It can even help to quickly determine the indications for surgery. Our proposed algorithm has the potential to revolutionize the diagnosis and management of nasal bone fractures, leading to improved patient outcomes and clinical efficiency. Further studies are required to validate these findings.

## Figures and Tables

**Table 1:** Baseline characteristics of patients.

Characteristics	Training Set (n = 2099)	Validation Set (n = 730)	Test Set (n = 315)	*p*-Value *
Age, mean ± SD	39 ± 19	38 ± 21	41 ± 20	0.112
Sex, n (%)				<0.001
Male	1328 (63.3)	458 (62.7)	176 (55.9)	
Female	771 (36.7)	272 (37.3)	139 (44.1)	
Label, n (%)				0.081
Fracture	1276 (60.8)	412 (56.4)	173 (54.9)	
Normal	823 (39.2)	318 (43.6)	142 (45.1)	

SD standard deviation. * *p*-values were calculated using Pearson’s Chi-squared test.

**Table 2:** Summary of the fracture detection AI model’s performance according to the labeling methods.

Labeling Methods	Accuracy	Precision	Sensitivity	F1 Score
Classification	67.09	67.90	65.80	66.80
Object detection	67.41	67.30	66.70	67.00
Segmentation	97.68	82.2	88.9	85.4

## Data Availability

The data from this study will be made available by the corresponding authors upon request. Due to privacy and ethical restrictions, the data is not publicly accessible.

## Abbreviations

The following abbreviations are used in this manuscript:

AI	Artificial intelligence
DCNN	Deep convolutional neural networks
CNN	Convolutional neural network
CT	Computed tomography
ROI	Region of interest
ROC	Receiver operating characteristic
NPV	Negative predictive value
AUC	Area under the curve
CAM	Class activation mapping
